# Identification of a major-effect QTL associated with pre-harvest sprouting in cucumber (*Cucumis sativus* L.) using the QTL-seq method

**DOI:** 10.1186/s12864-021-07548-8

**Published:** 2021-04-07

**Authors:** Mingming Cao, Shuju Li, Qiang Deng, Huizhe Wang, Ruihuan Yang

**Affiliations:** 1 State Key Laboratory of Vegetable Germplasm Innovation, Tianjin Key Laboratory of Vegetable Breeding Enterprise, Tianjin Kernel Cucumber Research Institute, Tianjin, 300192 China; 2 Institute of Cucumber Research, Tianjin Academy of Agricultural Sciences, Tianjin, 300192 China

**Keywords:** Cucumber, Pre-harvest sprouting, QTL-seq, *qPHS4.1*

## Abstract

**Background:**

Cucumber (*Cucumis sativus* L.) is cultivated worldwide, and it is essential to produce enough high-quality seeds to meet demand. Pre-harvest sprouting (PHS) in cucumber is a critical problem and causes serious damage to seed production and quality. Nevertheless, the genetic basis and molecular mechanisms underlying cucumber PHS remain unclear. QTL-seq is an efficient approach for rapid quantitative trait loci (QTL) identification that simultaneously takes advantage of bulked-segregant analysis (BSA) and whole-genome resequencing. In the present research, QTL-seq analysis was performed to identify QTLs associated with PHS in cucumber using an F_2_ segregating population.

**Results:**

Two QTLs that spanned 7.3 Mb on Chromosome 4 and 0.15 Mb on Chromosome 5 were identified by QTL-seq and named *qPHS4.1* and *qPHS5.1*, respectively. Subsequently, SNP and InDel markers selected from the candidate regions were used to refine the intervals using the extended F_2_ populations grown in the 2016 and 2017 seasons. Finally, *qPHS4.1* was narrowed to 0.53 Mb on chromosome 4 flanked by the markers SNP-16 and SNP-24 and was found to explain 19–22% of the phenotypic variation in cucumber PHS. These results reveal that *qPHS4.1* is a major-effect QTL associated with PHS in cucumber. Based on gene annotations and qRT-PCR expression analyses, *Csa4G622760* and *Csa4G622800* were proposed as the candidate genes.

**Conclusions:**

These results provide novel insights into the genetic mechanism controlling PHS in cucumber and highlight the potential for marker-assisted selection of PHS resistance breeding.

**Supplementary Information:**

The online version contains supplementary material available at 10.1186/s12864-021-07548-8.

## Introduction

Cucumber (*Cucumis sativus* L.) is an economically important vegetable globally. In 2018, cucumber was grown on 1,984,518 ha worldwide, and the cultivated area in China accounted for 52.72% of this area (www.fao.org/faostat/en). It is necessary to produce enough excellent-quality cucumber seeds to meet demand, especially in China. However, pre-harvest sprouting (PHS), also known as vivipary, a critical trait describing the untimely germination of seeds inside maternal fruits under certain conditions, severely decreases seed yields and quality [1]. Breeding for resistance to PHS would decrease the loss of usable seeds in cucumber.

In agriculture, it is widely accepted that PHS is a complex agronomic trait controlled by multiple genes or quantitative trait loci (QTLs) [2, 3]. PHS is tightly connected with seed dormancy which is characterized as the prevention of physiologically mature seeds from germinating under unfavorable environmental conditions [4, 5]. Low levels of seed dormancy lead to PHS [6], while excessive seed dormancy usually gives rise to PHS resistance but unfortunately causes undesirable results, such as nonuniform seedling establishment after sowing [7, 8]. Therefore, maintenance of the balance between seed dormancy and germination is critical.

Regarding the genetic and molecular basis of seed dormancy and PHS resistance, extensive QTLs or genes for this trait have been identified in cereal crops and other vegetables, such as rice (*Oryza sativa*), wheat (*Triticum aestivum*), maize (*Zea mays*), barley (*Hordeum vulgare*) and tomato (*Solanum lycopersicum*). To date, in rice, more than 165 QTLs associated with seed dormancy or PHS resistance and located on different chromosomes have been identified [9, 10]. Similar to rice, QTLs responsible for PHS identified in wheat, which has a much more complicated genome, were distributed on almost all of the chromosomes [11]. Among them, the major QTLs were detected mainly on chromosome 2B [12], 3AS [13], and 7B [14], while minor QTLs were detected on chromosomes 3B and 5A [13]. In barley, several QTLs associated with seed dormancy have been identified [15–17]. Among the QTLs, two QTLs, *SD1* and *SD2* on chromosome 5H, contributed the major effects on seed dormancy [18]. *SD1* was a major regulator of dormancy [19], and *SD2* was identified to prevent PHS [17]. However, to date, QTL genetic mapping for PHS in cucumber has not been reported.

Traditional QTL mapping requires a segregating population originating from two parents with extreme opposite traits and polymorphic markers linked to target genes. It is extremely time-consuming and labor-intensive to screen DNA markers and genotype individuals in the segregating populations [20]. Bulked-segregant analysis (BSA) is an effective method to rapidly identify polymorphic markers linked to traits of interest [21]. QTL-seq [22], a powerful new approach combining BSA and next-generation sequencing, is used for the rapid identification of QTLs. Recently, QTL-seq has been widely used in the detection of QTLs for many traits in various plants, including 100-seed weight trait in chickpea [23], branch angle in oilseed rape [24], fruit length in cucumber [25], stalk rot in maize [26], heat-tolerance and high-temperature stress response in tomato [27], and cooked grain elongation [28] and salt tolerance [29] in rice. Therefore, QTL-seq provides a convenient method for identifying key loci controlling PHS in cucumber.

Our previous studies have revealed that PHS was controlled by one major gene of additive-dominance effects plus additive-dominance polygene (D-1 model) via the method of mixed major-gene plus polygenes inheritance model [30]. However, the genetic mapping and QTL location have not been performed. In this paper, we performed QTL-Seq analysis using an F_2_ population derived from Q12 and P60, which are resistant and susceptible to PHS in cucumber, respectively. SNP and InDel markers generated from QTL-seq were developed to genotype all the individuals in the F_2_ population grown in two years. The major QTLs were refined, and annotated genes located in the associated regions were analyzed by quantitative RT-PCR. This study may have the potential for cucumber breeding of PHS resistance by marker-assisted selection (MAS) and gene cloning analysis.

## Results

### Phenotypic evaluation of PHS in cucumber

The seeds of the resistant parent Q12, susceptible parent P60, and their F_1_, F_2_ populations were sown directly into soil in the greenhouse on April 15 each year. For plant management, two female flowers were self-pollinated, and all the other female flowers and lateral branches were removed from each plant. The pollination date was recorded on labels hung on the peduncles of the fruits. The seeds in the cucumber fruits were harvested at 45 days after pollination (DAP), and the numbers of germinated seeds and total seeds were counted immediately. The PHS rate (%) was calculated as (germinated seeds/total seeds in fruit) × 100%. The average PHS rates of two cucumber fruits grown on the same plant were used for QTL analysis.

**Fig. 1:**
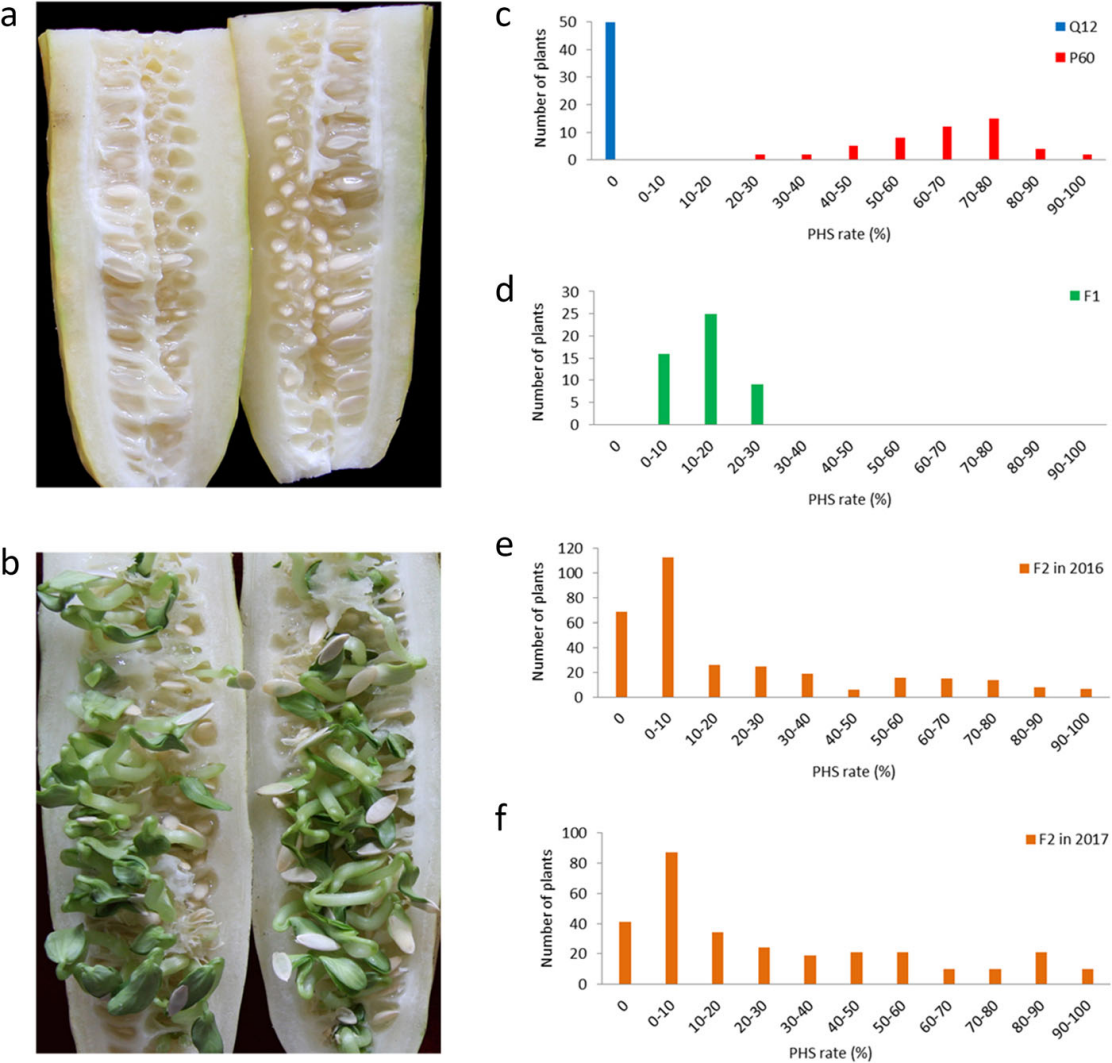
Pre-harvest sprouting (PHS) and its frequency distribution in the parental lines, F_1_ and F_2_ populations. **a**: Phenotype of Q12, resistant to PHS; **b**: Phenotype of P60, susceptible to PHS; **c**: Frequency distribution of PHS in the parental lines, Q12 and P60; **d**: Frequency distribution of PHS in the F_1_ generation grown in 2016; **e**: Frequency distribution of F_2_ population grown in 2016; **f**: Frequency distribution of F_2_ population grown in 2017

Phenotypic data of the PHS rate were collected from Q12, P60, and their F_1_, F_2_ populations (Additional file 1: Table S1). Q12 showed complete resistance to PHS; P60 displayed a wide range of variation for PHS (Fig. 1). The mean PHS rates of Q12, P60 and F_1_ progeny were 0, 64.97 and 13.88%, respectively. The PHS rates of the segregating mapping population of 328 F_2_ individuals grown in 2016 covered the full range from 0 to 100% (20.77% on average) and showed a skewed normal distribution (Fig. 1). The PHS rates of the 298 F_2_ individuals grown in 2017 showed a similar distribution to 2016. This phenotypic variation in the populations indicated that PHS is a quantitative trait controlled by a major-effect QTL.

### Pool construction and QTL-seq

Based on the phenotypic data of F_2_ individuals (Additional file 1: Table S1), 30 extremely resistant and 30 extremely susceptible individuals were selected from the F_2_ population grown in 2017 for the construction of the R- and S-pool, respectively. The PHS rate of each extreme F_2_ individual in the R-pool was 0%, and the PHS rate of extreme individuals in the S-pool ranged from 80 to 100%. Each DNA pool, along with the R-parent (Q12) and S-parent (P60), were subjected to whole-genome resequencing (WGRS) using the Illumina HiSeq4000 platform, and 36.83 Gb raw data was generated. The clean data were mapped to the cucumber reference genome (http://www.cucurbitgenomics.org/organism/2; Chinese long; V2) [31] using the BWA 0.7.10 (Burrows-Wheeler Aligner) software [32], and 36.55 Gb remained after trimming and adapter removal. A total of 6.83 Gb clean data (18.59X coverage) for Q12, 8.43 Gb (22.30X coverage) for P60, 10.37 Gb for the R-pool (28.06X coverage) and 10.92 Gb (30.54X coverage) S-pool was generated. Detailed information is listed in Table 1.

**Table 1:** Resequencing summary of the parental lines, R-pool and S-pool

Sample	Clean bases (Gb)	Total reads	Mapped reads	Rate of mapped reads(%)	Sequencing depth (X)	Genome coverage
Q12	6.83	45,507,344	38,958,916	85.61	18.59	98.75
P60	8.43	56,203,612	47,443,606	84.41	22.30	98.82
R-pool	10.37	69,130,804	61,513,704	88.98	28.06	98.99
S-pool	10.92	72,805,348	63,923,453	87.80	30.54	98.99

**Fig. 2:**
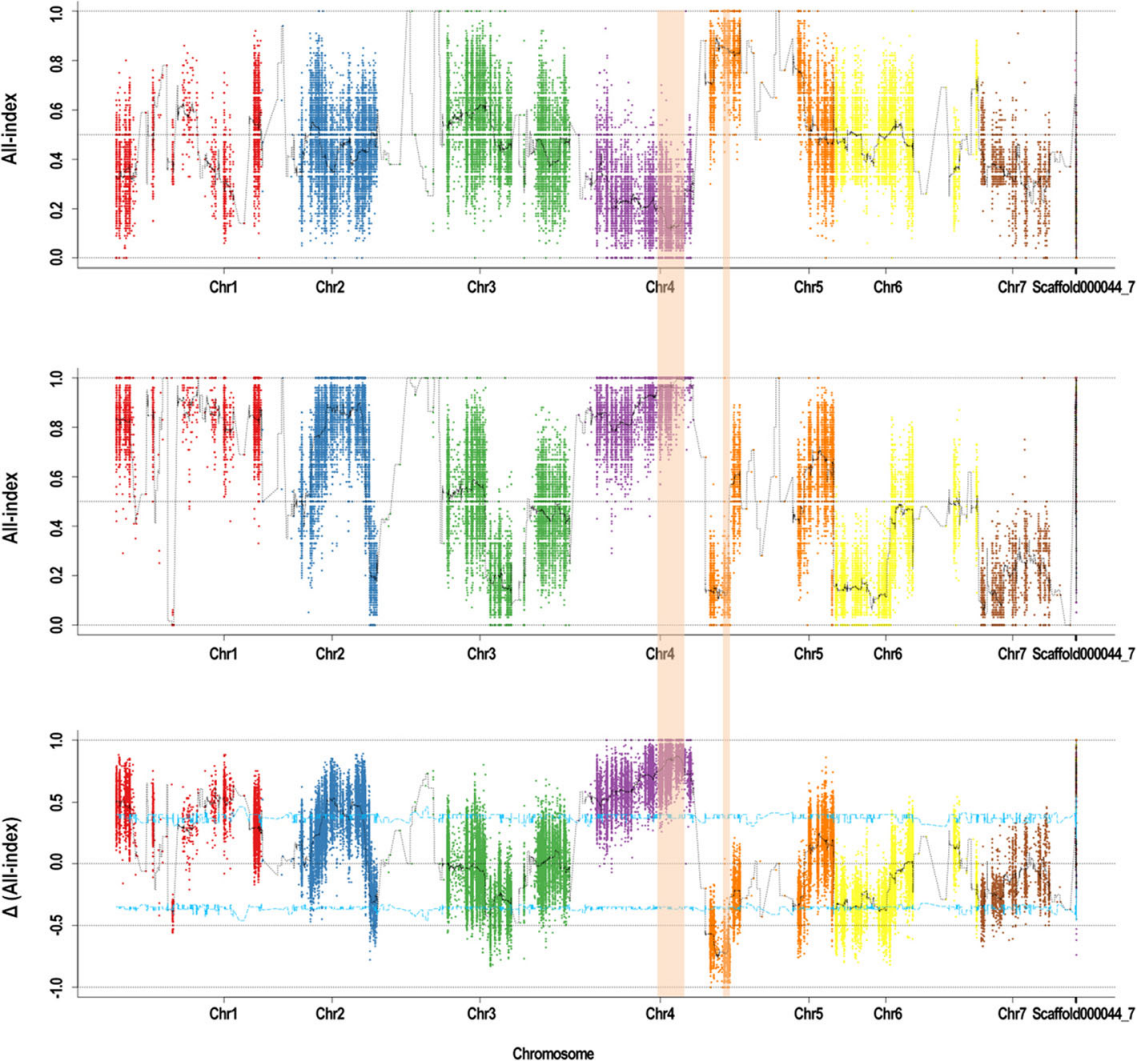
SNP/InDel-index Manhattan graphs of R-pool, S-pool and Δ (SNP/InDel-index) from QTL-seq approach for mapping the genomic regions controlling pre-harvest sprouting in cucumber. **a**: SNP/InDel-index plot of R-pool; **b**: SNP/InDel-index plot of S-pool; **c**: the Δ (SNP/InDel-index) plot of all chromosomes with the statistical confidence interval under the null hypothesis of no QTLs (blue line *P* = 0.05). The significant genomic regions on Chromosome 4 and 5 are highlighted in shaded color

Using GATK 3.8 software [33], a total of 62,504 SNPs and 18,646 InDel variants were detected between the two parents. The Δ (SNP/InDel-index) of the polymorphic loci between R-pool and S-pool was calculated based on the SNP/InDel-index in R-pool and S-pool. The sliding window approach was used, and SNP/InDel-index plotted graphs against the genomic positions for R-pool (Fig. 2a) and S-pool (Fig. 2b) were generated. After calculating, Δ (SNP/InDel-index) plotted graph was constructed (Fig. 2c). Two regions harboring high Δ (SNP/InDel-index) values exceeding the confidence interval and containing variations with SNP/InDel-index = ‘0’ or ‘1’ were examined and defined as the predicted regions associated with PHS. As a result, the SNP-index of predicted regions for R-pool and S-pool appeared as mirror images [22]. One of the regions spanned 7.3 Mb on chromosome 4, and the other region spanned 0.15 Mb on chromosome 5. We named these two predicted regions that were putatively associated with PHS in cucumber *qPHS4.1* and *qPHS5.1*, respectively. In *qPHS4.1*, several loci with the highest Δ (SNP/InDel-index) value equal to ‘1’ were detected. Conversely, *qPHS5.1* region was harboring loci with the lowest Δ (SNP/InDel-index) value equal to ‘-1’. These results indicated that the QTLs were associated with PHS in cucumber. *qPHS4.1* conferred a partial level of PHS resistance in the resistant donor Q12, while *qPHS5.1* provided partial resistance for the parent P60.

These two regions contained 443 SNPs and 124 InDels, of which 272 SNPs and 82 InDels were found to be intergenic, 70 SNPs and 19 InDels intronic, 4 SNPs synonymous, 6 SNPs nonsynonymous, 39 SNPs and 11 InDels in upstream and 47 SNPs and 10 InDels in downstream (Table 2). In *qPHS5.1*, there were only two InDels detected in upstream. The other variations were identified in *qPHS4.1*interval.

**Table 2:** Categorization of Detected Variations in *qPHS4.1* and *qPHS5.1*

Category		*qPHS4.1*		*qPHS5.1*	
		SNPs	InDels	SNPs	InDels
Exonic	Synonymous	4	0		
	Non-Synonymous	6	0		
	Non-Frameshift Insertion	–	1		
Intronic		70	19		
Upstream		39	9		2
Downstream		47	10		
Upstream/Downstream		5	1		
Intergenic		272	82		
transition		277	–		
transversion		166	–		
Insertion		–	62		
Deletion		–	62		
Total		443	124		

Based on the gene annotation via ANNOVAR (Version 2013Aug23) software [34], genes containing stop loss, stop gain or nonsynonymous mutations were preferentially selected as candidate genes (Additional file 2: Table S2) from the associated regions.

### Validation and narrowing down the associated region

**Fig. 3:**
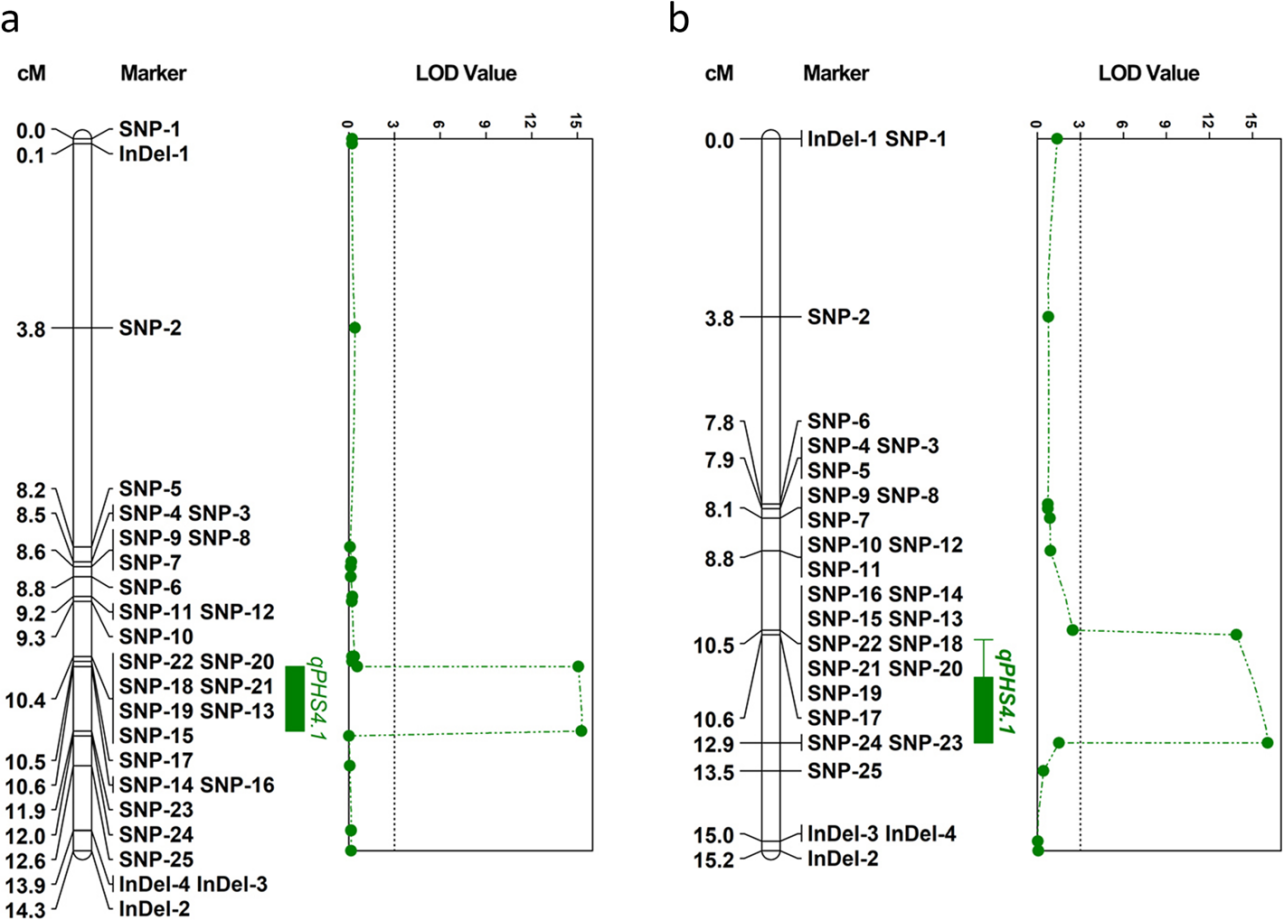
Fine mapping of the major-effect QTL*qPHS4.1*in cucumber using F_2_ populations grown in 2016 (**a**) and 2017 (**b**). SNP and InDel markers in candidate regions generated by QTL-seq were selected and genotyped in the 318 F_2_ individuals grown in 2016 and 298 F_2_ individuals grown in 2017. One major-effect QTL in the overlapping region was identified. The interval of *qPHS4.1* was narrowed down to 0.53 Mb on Chromosome 4

To verify the results detected by QTL-seq and narrow down the candidate intervals, a traditional QTL mapping method was used. We genotyped all F_2_ individuals grown in 2016 and 2017 for 62 SNP and/or InDel markers selected from the *qPHS4.1* and *qPHS5.1* intervals, respectively. Finally, twenty-nine markers in *qPHS4.1* were accurately genotyped and applied to construct the local genetic linkage maps by JoinMap 4.0 software [35]. Two InDel markers on Chromosome 5 were unmapped. After calculation by MapQTL version 6 software [36], two loci with LOD scores over the threshold, SNP-16 and SNP-23, were found by using the 2016 F_2_ population. As shown in Table 3, the peak LOD scores of SNP-16 and SNP-23 were 15.07 and 15.28, respectively. This interval explained 19.6–19.8% of the phenotypic variation in PHS. In the 2017 F_2_ population, two peak SNP loci, SNP-17 (LOD = 13.89) and SNP-24 (LOD = 16.06), were detected (Table 3, Additional file 3: Table S3). The interval explained 19.3–22.0% of the phenotypic variation in PHS. By taking the overlapping regions into account, these results reduced the candidate genomic interval associated with *qPHS4.1* from 7.3 Mb to the 0.53 Mb flanked by the markers SNP-17 to SNP-23 on chromosome 4 in cucumber (Fig. 3).

**Table 3:** LOD Values, Additive Effects, and Variance Explained for the Significant Loci Associated with pre-harvest sprouting in Cucumber

Year	The SNP markers	Physical position on Chromosome 4 (bp)	Interval(Mb)	LOD^a^	Additive effect^b^	Dominance	Variance explained(%)^c^
2016	SNP-16	19,973,741	0.53	15.07	−0.136391	−0.0594552	19.6
	SNP-23	20,505,510		15.28	−0.141843	−0.0345112	19.8
2017	SNP-17	19,973,782	0.55	13.89	−0.159806	−0.0408643	19.3
	SNP-24	20,521,004		16.06	−0.182258	0.0193450	22.0

^a^Peak LOD score of the QTL. ^b^Additive or dominant effect of the SNPs. ^c^Percentage of variance explained by the QTL peak

### Gene annotation and expression analysis of candidate genes

**Fig. 4:**
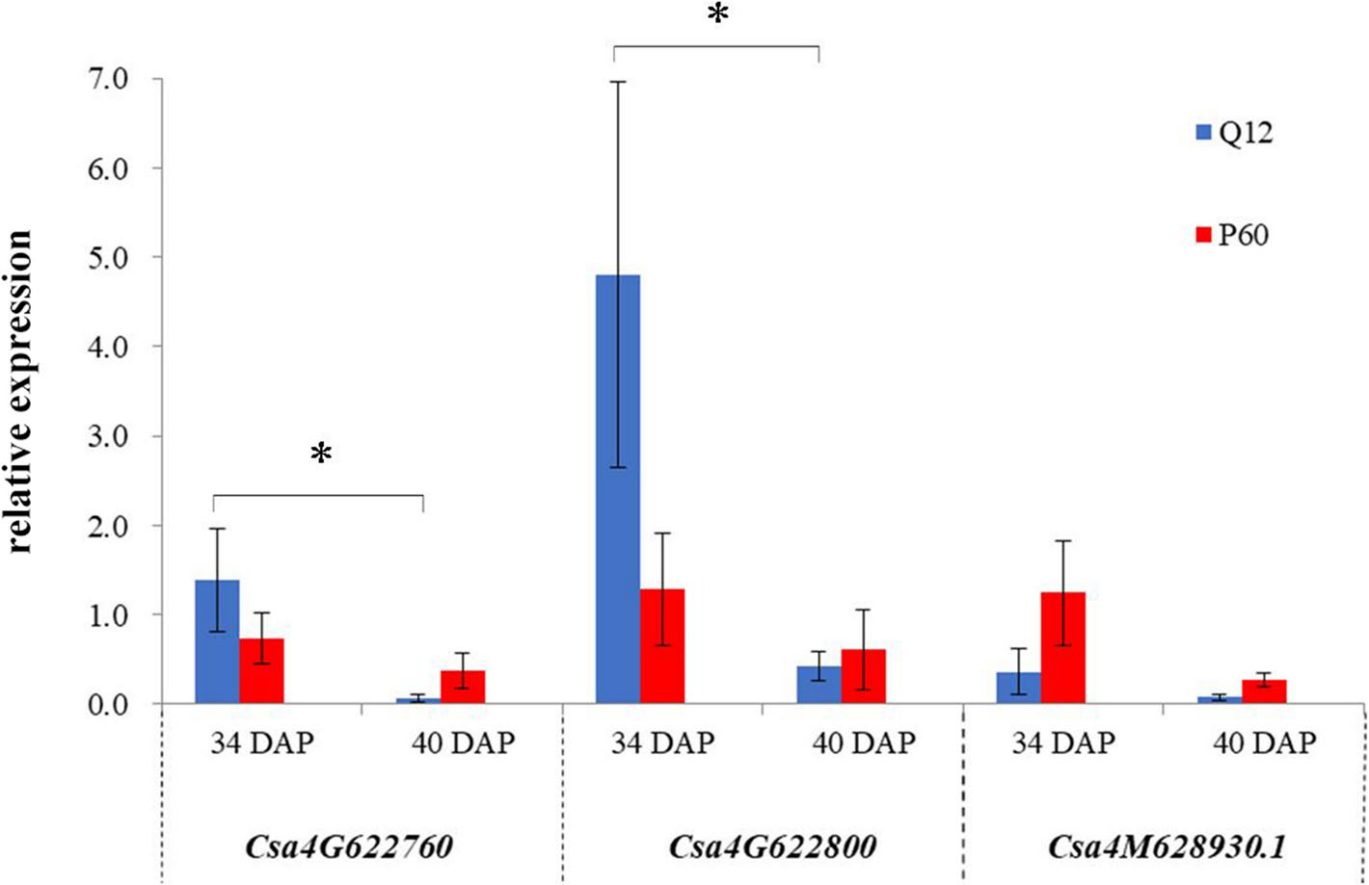
The relative quantitative expression analysis of the predicted genes in cucumber cavity flesh tissue of Q12 and P60. The blue bars represent Q12, the red bars represent P60. 34 DAP indicates the relative gene-expression levels in the cucumber cavity flesh tissue sampled from cucumber fruits at 34 days after pollination (DAP), at which point the seeds had not germinated in the cucumber cavities. 40 DAP indicates the relative gene-expression levels in the cucumber fruits at 40 days after pollination, at which point the seeds had germinated in those cucumbers that were susceptible to pre-harvest sprouting. Data are the means of three biological and technical replicates ± the standard error. * *P* < 0.05 in the *t*-test

On the basis of the gene annotations, within the *qPHS4.1* region, *Csa4G622760*, *Csa4G622800* and *Csa4M628930.1* (Table 4), in which nonsynonymous or upstream mutations occurred, were selected as candidate genes for further analysis. The relative expression levels of the candidate genes in seed cavity flesh tissues between Q12 and P60 were examined by Real-time Quantitative PCR (qRT-PCR) at 34 DAP (PHS not occurred) and 40 DAP (PHS occurred) stages, as shown in Fig. 4. The expression level of *Csa4G622760*, which is predicted to encode a chalcone isomerase-like protein, was 1.9-fold higher in Q12 than in P60 at the 34 DAP stage. However, its expression level was 5.4-fold lower in Q12 than in P60 at 40 DAP. This indicated that the expression level of the *Csa4G622760* gene significantly decreased, by approximately 20-fold, from 34 DAP to 40 DAP in Q12 but was only 2-fold down-regulated in P60. The *Csa4G622800* gene is annotated as a peptide methionine sulfoxide reductase msrB. Its expression level was 3.7-fold higher in Q12 than in P60 at the 34 DAP stage. At the 40 DAP stage, the expression level was down-regulated 11.2-fold in Q12 and 2.1-fold in P60. Gene expression of *Csa4G622800* also decreased significantly. *Csa4M628930.1* is a putative ERI1 exoribonuclease 3 protein. At 34 DAP, the expression level in P60 was 3.43-fold higher than that in Q12. From 34 DAP to 40 DAP, gene expression decreased approximately 4.6-fold in both parental lines. At 40 DAP, the expression level in P60 was 3.41-fold higher than that in Q12. The expression pattern did not show significant differences.

**Table 4:** Candidate Genes Underlying *qPHS4.1* Control of Preharvest Sprouting in Cucumber

Gene ID	SNP location	SNP locus	Physical position (bp)	Mutation		Functional prediction
				Q12	P60	
*Csa4G622760*	upstream	SNP-14	19,973,692	G	T	Chalcone isomerase-like protein
	upstream	SNP-15	19,973,724	C	A	
	upstream	SNP-16	19,973,741	T	C	
	upstream	SNP-17	19,973,782	A	T	
*Csa4G622800*	upstream	SNP-18	19,995,077	A	G	Peptide methionine sulfoxide reductase msrB
	upstream	SNP-19	19,995,107	G	A	
	upstream	SNP-20	19,995,109	C	G	
	upstream	SNP-21	19,995,123	A	C	
	upstream	SNP-22	19,995,137	C	A	
*Csa4M628930.1*	nonsynonymous	SNP-23	20,505,510	T	C	ERI1 exoribonuclease 3

Taken together, these data show that the expression levels of the three genes were both down-regulated in Q12 and P60 with increasing ripeness of cucumber fruits. The *Csa4M628930.1* gene showed a different expression pattern from that of *Csa4G622760* and *Csa4G622800*. *Csa4G622760* and *Csa4G622800* gene expression levels significantly decreased (*p* < 0.05) in Q12 but decreased slightly in P60 from the 34 DAP stage to 40 DAP stage. These results suggested that *Csa4G622760* and *Csa4G622800* gene expression levels were higher in resistant cucumbers than in susceptible cucumbers before PHS occurred. Subsequently, accompanying the occurrence of PHS, its gene expression levels decreased significantly in resistant cucumbers compared to susceptible cucumbers. Therefore, we hypothesized that *Csa4G622760* and *Csa4G622800* are possible candidate genes involved in PHS in cucumber, but further functional analysis of these genes needs to be conducted.

## Discussion

In cucumber and other seed-bearing crops, pre-harvest sprouting (PHS) is a critical problem that causes devastating losses to seed yields and quality [1] and widely limits seed dispersal. To promote the process of cucumber PHS resistance breeding, it is greatly important to identify key loci controlling PHS resistance and develop molecular markers for marker-assisted selection (MAS). In cereal crops, including wheat, rice, maize and barley, PHS is a very popular research topic, and the investigation of genetic mapping and molecular mechanisms underlying PHS is extensive and intensive. However, unfortunately, few published studies have focused on the PHS trait in cucumber [32]. In this study, we identified two QTLs associated with PHS by a QTL-seq approach in the F_2_ population derived from the two parents Q12 and P60, which showed opposite extremes of PHS phenotypes. Q12 is a typical resistant line in which PHS never occurs in favorable environments, while PHS occurs in the P60 line (Fig. 1). The frequency distribution of PHS in P60 was normal. Subsequently, in the F_2_ population, the frequency distribution was skewed normal rather than normal (Fig. 1), suggesting that PHS was a quantitatively inherited trait in cucumber and controlled by a major-effect QTL. This is consistent with our previous research on the inheritance of PHS.

The application of high-throughput next-generation sequencing technology promotes the development of rapid molecular marker discovery and physical map construction. QTL-seq is a new method that combines next-generation sequencing with BSA for the rapid detection of QTLs and links molecular markers associated with traits of interest. It was first developed by Takagi et al. and applied in rice [22]. Since that time, QTL-seq has been successfully used in many species [23–29]. However, the candidate regions generated from QTL-Seq are often too rough or too broad, and additional QTL analysis performed by traditional methods is necessary to refine gene locations and narrow chromosomal intervals. In the present study, a QTL-seq approach was performed in the F_2_ population grown in 2017. Two QTLs associated with PHS, *qPHS4.1* and *qPHS5.1*, were initially identified, which spanned 7.3 Mb on chromosome 4 and 0.15 Mb on chromosome 5, respectively. The predicted regions in R-pool and S-pool appeared as mirror images [22] in Fig. 2. These results confirmed that *qPHS4.1* was derived from the resistant donor Q12 and *qPHS5.1* provided PHS resistance for P60. However, P60 was identified to be a susceptible genotype to PHS. Therefore, *qPHS5.1* could be a putative minor-effect QTL for PHS.

And then, traditional QTL mapping methods were conducted to validate and narrow down the candidate regions. The phenotype identification and QTL mapping using the extended F_2_ population grown in 2016 was consistent with the findings from the 2017 season, which indicated that the experimental results were reliable and accurate. Subsequently, the regions from the two seasons were found to overlap. Therefore, *qPHS4.1* was refined and narrowed down to 0.53 Mb on Chromosome 4. Unfortunately, *qPHS5.1* was unmapped by JoinMap 4.0 and MapQTL version 6 software. This result demonstrated that *qPHS4.1* was a major-effect QTL controlling PHS in cucumber and *qPHS5.1* was a merely minor-effect QTL. We supposed that only two InDel markers detected from QTL-seq were used in the validation. We need to develop more molecular markers to further analyze the minor-effect involved in *qPHS5.1* controlling PHS. As a major-effect QTL, *qPHS4.1* was identified to explain about 20% of the phenotypic variation. The available tightly linked markers in *qPHS4.1* can be used in MAS to promote the breeding process. We propose the introgression of *qPHS4.1* could provide a partial level of PHS resistance for a susceptible background genotype and decrease the PHS rate of susceptible cucumber lines in a certain extent.

Functional annotation of the *qPHS4.1* region, a total of 39 candidate genes was identified by ANNOVAR software. Based on gene expression analysis by qRT-PCR, two genes, *Csa4G622760* and *Csa4G622800,* containing upstream polymorphic SNPs, were considered candidate PHS regulating genes in cucumber (Fig. 4). The *Csa4G622760* gene is predicted to encode a chalcone isomerase-like protein that catalyzes the biosynthesis of flavonoids and secondary metabolism in plants [37]. Flavonoids are important secondary metabolites found in various plant tissues, such as leaves, flowers, fruits and seeds. In Arabidopsis, overexpression of the *chalcone isomerase-like* gene increased the accumulation of proanthocyanidin and flavonol, which are flavonoids, while loss of function of the *chalcone isomerase-like* gene led to a strong reduction in proanthocyanidin and flavonol levels and influenced the seed phenotype [38]. However, the correlation between flavonoids and PHS in cucumber is unclear. The *Csa4G622800* gene is predicted to encode the peptide methionine sulfoxide reductase msrB. In the promoters of methionine sulfoxide reductase genes, *cis*-regulatory elements were found from Arabidopsis, poplar and rice [39, 40]. Methionine sulfoxide reductase can play protective roles in redox homeostasis in plant growth, including seed development [41, 42]. In plant seeds, methionine sulfoxide reductase plays a decisive role in the establishment and preservation of seed longevity [43]. Higher activity of this enzyme leads to better preservation of the seeds and higher germination capacity [43]. In our research, the SNPs were identified in the promoter of *Csa4G622800* gene. We consider that the mutations in promoter of *Csa4G622800* gene would putatively alter the gene-expression levels and then affect the development and germination of seeds in cucumber fruits. However, further experiments need to be performed to test the functionality of the candidate genes in the genetic mechanisms of cucumber PHS.

In some cereal crops, e.g., wheat, rice, barley, etc., extensive QTLs and numerous genes associated with seed dormancy and PHS have been reported. However, only one major-effect QTL in cucumber was identified in this study. In contrast to cereal crops, cucumber seeds are surrounded by flesh tissues in seed cavities, in which the water content is higher than 95%. Therefore, PHS in cucumber is less likely to be influenced by the humidity of the environment. Cucumber PHS is a very specific and interesting trait, and the molecular mechanisms underlying PHS need further study.

## Conclusion

In this study, two QTLs associated with PHS in cucumber were detected using QTL-seq approach. The major-effect QTL *qPHS4.1* was refined to 0.53 Mb on chromosome 4. Based on the gene annotation and qRT-PCR analysis, two genes located in *qPHS4.1* were proposed to be the candidate genes associated with cucumber PHS. To our knowledge, this is the first report on the identification of QTLs associated with PHS trait in cucumber. This study provides novel insights into the genetic mechanism controlling PHS in cucumber and highlights the potential for PHS resistance MAS breeding.

## Materials and methods

### Plant materials and phenotypic evaluation

The high-generation inbred cucumber lines Q12 (North China fresh market cucumber, derived from Chinese commercial variety ‘Jinyan No.4’ crossed with ‘Sipingcigua’, PHS resistant, P_1_) and P60 (North China fresh market cucumber, generated from a Chinese commercial variety ‘YuanFengYuan No.6’, PHS susceptible, P_2_) were crossed to obtain F_1_. F_1_ plants were self-pollinated to generate an F_2_ segregating population. P_1_ and P_2_ populations were evaluated for PHS in the experimental farm of the Tianjin Kernel Cucumber Research Institute (Tianjin, China) in 2016. Significance test was conducted between P_1_ and P_2_ populations. The F_2_ population was evaluated in 2016 (328 plants) and 2017 (299 plants) seasons. All the plants were grown in greenhouse conditions under whole-day light exposure. The day/night temperature in the greenhouse was controlled at 28–35 °C/15–26 °C.

### Pool construction and whole-genome re-sequencing

The genomic DNA of Q12, P60 and F_2_ individuals was extracted from seedling leaves using a Quick Prep Plant Genome DNA Kit (HUALIKEXI, Tianjin, China). Q12 and P60 genomic DNA were used to construct the P_1_ pool and P_2_ pool. Based on the phenotype data of F_2_ individuals grown in the 2017 season (Additional file 1: Table S1), 30 extreme resistant plants and 30 extreme susceptible plants were selected to construct a resistant pool (R-pool) and susceptible pool (S-pool), respectively. Equal amounts of DNA from the selected individuals were mixed and subsequently processed to generate sequencing libraries using the TruSeq Nano DNA HT Sample preparation Kit (Illumina Inc., United States) by Novogene Co., Ltd. (http://www.novogene.com/). According to the protocol, briefly, DNA samples were randomly fragmented by sonication to a size of 350 bp, then DNA fragments were end polished, A-tailed, and ligated with the full-length adapter for Illumina sequencing with further PCR amplification. PCR products were purified (AMPure XP system) and libraries were analyzed for size distribution by Agilent2100 Bioanalyzer and quantified using real-time PCR [44]. These libraries were resequenced and 150 bp paired-end reads were generated with insert size around 350 bp using the Illumina HiSeq4000 platform (Illumina Inc., United States) by Novogene Co., Ltd. (http://www.novogene.com/).

### QTL-seq

The raw sequencing data were filtered to get high-quality clean reads by removing the reads with ≥10% unidentified nucleotides, removing the reads with > 50% bases having phred quality < 5 and the reads with > 10 nucleotides aligned to the adapter. The clean reads obtained from four pools were aligned to the cucumber reference genome (Chinese long; V2) [31] using the BWA 0.7.10 (Burrows-Wheeler Aligner) software [32]. Variant calling was performed for the samples by using the Unified Genotyper function in GATK 3.8 software [33]. To determine the genomic regions associated with PHS, we calculated the SNP/InDel-index and Δ (SNP/InDel-index) to locate the QTLs. The SNP/InDel-index refers to the proportion of reads carrying a SNP/InDel different from the reference reads of either parent. The Δ (SNP/InDel-index) of each locus was determined based on the difference in the SNP/InDel-index between the R-pool and S-pool. To eliminate background interference, we filtered out all loci with an SNP/InDel-index of less than 0.3 [22]. Using the slicing window method with a 1 Mb window size and 1 kb increment, the average SNP/InDel-index of loci in a given genomic interval was calculated. The SNP/InDel-index of the R-pool and S-pool and the corresponding Δ (SNP/InDel-index) in the slicing window were plotted in a graph to generate SNP/InDel-index plots. We calculated statistical confidence intervals of Δ (SNP/InDel-index) for all SNP and InDel loci with a given read depth under the null hypothesis of no QTL, following the detail procedures of Takagi et al. [22]. The confidence intervals of Δ (SNP/InDel -index) were defined to be 95% (*p* = 0.05). By examining the Δ (SNP/InDel-index), the candidate genomic regions harboring high average Δ (SNP/InDel-index) values exceeding the confidence intervals and containing variations with SNP/InDel-index = ‘0’ or ‘1’ were defined as predicted regions for association with PHS. SNP/InDel-index was equal to ‘0’ or ‘1’ when the candidate variations in pools were entirely from P_1_ or P_2_, respectively, and the corresponding Δ (SNP/InDel-index) was equal to ‘-1’ or ‘1’.

The ANNOVAR (Version 2013Aug23) software was used to annotate the candidate genes in the regions [34].

### Genotyping, regional linkage mapping and QTL analysis

To verify the candidate SNP and InDel markers and narrow down the regions identified by QTL-seq, significant SNPs and InDels in the candidate regions were first selected and validated in the two parents and their F_1_ plants. Then, polymorphic SNP and InDel markers were used to genotype the extended F_2_ individuals sown in the 2016 season and 2017 season. This validation was performed using Hi-SNP high-throughput genotyping method (Shanghai Biowing Applied Biotechnology CO. LTD, Shanghai, China). The specific multiplex PCR primers of the markers were designed by Primer 3 online software (http://frodo.wi.mit.edu/, Version 0.4.0) based on the cucumber reference genome (Chinese long; V2) [31] (listed in Additional file 4: Table S4). Multiplex PCR and high-throughput sequencing genotyping were performed as previously described [45, 46]. Based on the genotypes of significant SNPs and InDels in candidate regions of F_2_ individuals sown in 2016 (328 plants) and 2017 (299 plants), regional linkage maps were constructed using JoinMap 4.0 software [35] with the maximum likelihood mapping algorithm and Kosambi mapping function [47], respectively. According to the phenotyping datasets of the F_2_ individuals, QTL analysis was performed by the software MapQTL version 6 [36]. The “MQM mapping” algorithm with an LOD threshold score of > 3.0 was used to perform the calculation. The output logarithm of odds (LOD) scores were plotted along the genetic distances of the markers analyzed.

### Candidate gene annotation

According to the further narrowed region of the QTLs, effective SNPs or InDels associated with PHS were identified. Based on the Cucurbit Genomics Database (http://www.icugi.org/cgi-bin/ICuGI/index.cgi), the functions of candidate PHS-associated genes that contained non-synonymous or upstream/downstream variations were predicted.

### Gene expression analysis by qRT-PCR

We used qRT-PCR to investigate the relative expression levels of the candidate genes between the two parents. The cucumber cavity flesh tissues surrounding the seeds at 34 DAP and 40 DAP were sampled, and RNA was extracted using TRNzol Universal Reagent following the manufacturer’s protocol (TIANGEN, Beijing, China). The RNA quality was evaluated by agarose gel electrophoresis. cDNA was synthesized using a RevertAid First Strand cDNA Synthesis Kit (Thermo Fisher Scientific Inc., MA, USA). Primers for candidate genes were designed by Primer 3 and synthesized by Sangon Biotech (Shanghai) Co., Ltd. (Sangon, Shanghai, China). Details of the primer sequences are presented in Table 5. qRT-PCR was conducted by using TB Green Premix Ex Taq II (Takara Bio Inc., Dalian, China) on a real-time PCR system (Step One Plus; Applied Biosystems). The qRT-PCR conditions were set as follows: 95 °C for 30 s; followed by 40 cycles of 95 °C for 5 s and then 60 °C for 30 s; and then denaturation at 95 °C for 15 s, 60 °C for 60 s, a temperature increase of 0.3 °C per 15 s, and finally 95 °C for 15 s. The *tubulin* gene (GenBank ID: AF044573.1) was used as the reference gene for normalization of the relative expression of the candidate genes. The relative expression levels of the target genes were calculated using the 2^−ΔΔCt^ method [48]. The experiments were conducted with three biological and technical replicates. A Student’s *t*-test was used to check the significant differences in expression levels among the samples.

**Table 5:** qRT-PCR primers for candidate genes and reference gene (*Tubulin*)

Gene ID	F	R	Size (bp)
*Csa4G622760*	TAACTCTGCCAGGCTGCTCAAC	GTCTCGGACAAGAACAATCTGTAAAG	242
*Csa4G622800*	GCATCAAAGAGGCTGGCACC	TATCCCTGCCGTTTTGGTGTTC	152
*Csa4M628930.1*	GGAGTTGACCGTGTCTGGC	TGATGTGGGACCTGAGTTT	173
*Tubulin*	GCAAGGAAGATGCTGCCAATA	TCCATAGTCAACAGACAAACGCTC	203

## Supplementary Information

**Additional file 1: Table S1**. Phenotypic evaluation of pre-harvest sprouting trait for parents and their F_1_ and F_2_ populations.

**Additional file 2: Table S2**. Candidate genes by annotation and loci generated from QTL-seq.

**Additional file 3: Table S3**. Physical position, genetic distance, LOD values and variations explained generated from software MapQTL version 6.

**Additional file 4: Table S4**. Detailed multiplex PCR Primers of the Markers.

## Data Availability

The raw datasets are stored in NCBI. The accessions of the datasets are SRR13637896, SRR13637895, SRR13637894 and SRR13637893. The analyzed data during this study are included in this published article and its supplementary information files.
