# Supplementary material for: Identification of a major-effect QTL associated with pre-harvest sprouting in cucumber (Cucumis sativus L.) using the QTL-seq method
Source: BMC Genomics. 2021 Apr 7;22:249. doi: 10.1186/s12864-021-07548-8 (PMC8028694; doi:10.1186/s12864-021-07548-8)
Supplement: Supplementary file 4 — Additional file 4: Table S4. Detailed multiplex PCR Primers of the Markers. [file 12864_2021_7548_MOESM4_ESM.docx]

**Identification of a Major-effect QTL Associated with Pre-harvest Sprouting in Cucumber (*Cucumis sativus* L.) Using the QTL-Seq Method**

**Mingming Cao^1^, Shuju Li^1*^, Qiang Deng^1^, Huizhe Wang^2^, Ruihuan Yang^2^**

^1^ State Key Laboratory of Vegetable Germplasm Innovation, Tianjin Key Laboratory of Vegetable Breeding Enterprise, Tianjin Kernel Cucumber Research Institute, Tianjin 300192, China

^2^Institute of Cucumber Research, Tianjin Academy of Agricultural Sciences, Tianjin 300192, China

*** Correspondence:**

Shuju Li

lishuju1964@126.com

Email address :

Mingming Cao: caoming2013@126.com

Shuju Li: lishuju1964@126.com

Qiang Deng: dengqiang022@126.com

Huizhe Wang: wanghuizhe@126.com

Ruihuan Yang: yruihuan@126.com

Table S4 The Physical Position and Primers of the Markers

| Locus | Physical position (bp) on Chr4 | F-primer | R-primer |
| --- | --- | --- | --- |
| SNP-1 | 13786976 | ACAACTTTGGTCATTAAAGACACC | TGGCTAATTTTATACTTCTTTTCATGC |
| InDel-1 | 13786987 |  |  |
| SNP-2 | 17143696 | ACTGGAGCAGATGACAATATATCC | AACAACATGAGAGTGGAAAAGAAG |
| SNP-3 | 18251895 | CGAGAAAGATTGCACTAAAATGTC | CTCGATCGAAGAATCTCTACTTTG |
| SNP-4 | 18251951 |  |  |
| SNP-5 | 18251971 |  |  |
| SNP-6 | 18251973 |  |  |
| SNP-7 | 18601010 | CTTTCTCCAAAGAACCTCTTTCTC | TTTCACCTTCCAATTTCAATCCTC |
| SNP-8 | 18605011 | TTGTTATTAGGATGCTTCCCTTTG | GCCAACATCAGACTAAGAATCATC |
| SNP-9 | 18614772 | TGTAGGTAACAAGTACATGGGTAG | CATTCGCTGATTAAAATGAAGCAG |
| SNP-10 | 18937823 | TAGACTCCATGTCACTACATTTTC | CTCCACTAAAAACTTATAGCCACG |
| SNP-11 | 18938019 | GCTCTCTGATTGTCGTCTGTTACG | CTAATGGACCTAGAGATCACAAGCTAC |
| SNP-12 | 18938031 |  |  |
| SNP-13 | 19973681 | GCATTCTATCCAATATGGTACTTCC | GGTTTCTTCCTCATCCATACTTATC |
| SNP-14 | 19973692 |  |  |
| SNP-15 | 19973724 |  |  |
| SNP-16 | 19973741 |  |  |
| SNP-17 | 19973782 |  |  |
| SNP-18 | 19995077 | TTCATGATTCTGAAATAAAACAGC | TGATGACTTTATCTCGAAAACACC |
| SNP-19 | 19995107 |  |  |
| SNP-20 | 19995109 |  |  |
| SNP-21 | 19995123 |  |  |
| SNP-22 | 19995137 |  |  |
| SNP-23 | 20505510 | AATGTGTTTCTCTCTTGACAACAG | TTCTCTCTTTACTTCTACCAGTGG |
| SNP-24 | 20521004 | TGTACCTATAAATCTTGGAACCTTC | TCAGGAGCTTGCCTATTTAAAAAC |
| SNP-25 | 20791425 | TAAATCTAATATTATTATCATGTTGTTGTG | GTTCTTTCCCAGCCAAAATTTG |
| InDel-2 | 21075353 | GTATCAACAATATCAATGTAATGGTTG | CTAAGTGATATAAATGTTGATAGCATG |
| InDel-3 | 21075917 | GACTATTTTATTCATCGGGCTCTC | AAATGACAATTTTGCCCCTTAAAC |
| InDel-4 | 21079500 | ACCATTGTGTAAATGTATGAAATTTCC | TTTGCAAATACCAATGTAACGTTC |
